# Dispersibility of the Pale Grass Blue Butterfly *Zizeeria maha* (Lepidoptera: Lycaenidae) Revealed by One-Individual Tracking in the Field: Quantitative Comparisons between Subspecies and between Sexes

**DOI:** 10.3390/insects11020122

**Published:** 2020-02-14

**Authors:** Atsuki Hiyama, Joji M. Otaki

**Affiliations:** 1 The BCPH Unit of Molecular Physiology, Department of Chemistry, Biology and Marine Science, Faculty of Science, University of the Ryukyus, Okinawa 903-0213, Japan; 2 Laboratory of Conservation Ecology, Faculty of Science and Engineering, Chuo University, Tokyo 112-8551, Japan; 3 Japan Butterfly Conservation Society, Tokyo 140-0014, Japan

**Keywords:** butterfly behavior, dispersibility, Fukushima nuclear accident, indicator species, one-individual tracking, pale grass blue butterfly, subspecies, *Zizeeria maha*

## Abstract

The pale grass blue butterfly *Zizeeria maha* (Lepidoptera: Lycaenidae) has been used as an environmental indicator species for radioactive pollution after the Fukushima nuclear accident. Here, based on the one-individual tracking method in the field, we examined dispersal-associated and other behavioral traits of this butterfly, focusing on two subspecies, *Z. maha argia* in mainland Japan and *Z. maha okinawana* in Okinawa. The accumulated distances in the adult lifespan were 18.9 km and 38.2 km in mainland and Okinawa males, respectively, and 15.0 km and 7.8 km in mainland and Okinawa females, respectively. However, the mean distance from the starting point was only 24.2 m and 21.1 m in the mainland and Okinawa males, respectively, and 13.7 m and 7.4 m in the mainland and Okinawa females, respectively. Some quantitative differences in resting and feeding were found between subspecies and between sexes. The ARIMA (autoregressive integrated moving average) model indicated that the dispersal distance was 52.3 m (99% confidence interval value of 706.6 m) from the starting point in mainland males. These results support the idea that despite some behavioral differences, both subspecies of this butterfly are suitable as an environmental indicator because of the small dispersal ranges.

## 1. Introduction

The dispersal range of an organism (the mobility of an individual in its lifetime) has crucial importance in ecology, population biology, evolutionary biology, and environmental sciences because the dispersal range of a given individual defines the population structures of that species [1,2,3,4,5]. Dispersal trait polymorphisms among individuals within a population are responsible for the isolation (fragmentation) and unification of populations [2,6]. The morphological and physiological changes in dispersers observed in some insect populations appear to be associated with current human-induced global environmental changes [6,7,8]. As dispersal traits are heritable [9], it is likely that variations of this trait directly affect the course of evolution [3,7,10]. It has been considered that the dispersal of a given individual regulates the networks of available resources for the population [1]. Therefore, information about dispersal-associated traits of species in the field is important for understanding dynamic ecosystems and is essential for policy makers to propose conservation strategies for endangered populations and species [1].

To study ecological dynamics in an environment, it is customary to focus on a few surrogate species that can represent an aspect of the biological community [11]. To choose a candidate indicator species, understanding the dispersal trait, in addition to the other ecological traits of the species, is essential, although there are several criteria for an indicator species [11]. One study [12] described 10 indispensable criteria for an indicator organism as follows: (1) easy species identification; (2) amenability to field survey; (3) independence from weather and time; (4) reasonably low survey cost; (5) wide distribution range; (6) small dispersal range; (7) high population density; (8) easy quantification; (9) monophagous nature; and (10) practicality and easiness for citizens including children to survey. Although these criteria are not applicable to the evaluation of the specificity of the environment, which is the role of, for example, keystone species, these criteria are useful for identifying indicator species that could reflect the biotic safety of the environment [12].

Among Japanese butterflies, the pale grass blue butterfly *Zizeeria maha* (Koller, 1844) is probably one of the most suitable species that fully meets these criteria at the species level. The pale grass blue butterfly is a small lycaenid butterfly common throughout Japan (except Hokkaido) [13,14,15,16]. This species is one of the most abundant and easy-to-find species among Japanese butterflies [17]. In Japan, this species is divided into two subspecies by the Watase line (the Tokara gap); *Z. maha argia* is found north of the line (the mainland subspecies) and *Z. maha okinawana* is found south of the line (the Okinawa subspecies). It has been shown that this species can be found and captured irrespective of weather conditions [18]. Larvae of this species are monophagous, feeding only on the host plant *Oxalis corniculata* (and its very closely related species) [13,14]. For these reasons, *Z. maha* is a suitable indicator for the safety of the environment.

Reflecting these facts, this butterfly has been used as an indicator species. For example, this butterfly was used for a risk assessment of transgenic maize [19]. After the establishment of an efficient rearing method [20], a series of reports evaluated the risks of radioactive materials spread in the environment caused by the collapse of the Fukushima Dai-ichi Nuclear Power Plant using this butterfly [21,22,23,24,25,26,27,28,29,30,31,32,33,34,35,36]. An increase in aberrant individuals of the pale grass blue butterfly in polluted localities has been described in these studies, where collected individuals in a given locality were assumed to be native to that locality. In other words, this butterfly was assumed not to disperse for long distances. Although this assumption is reasonable, considering the small body size and weak flying ability of this butterfly, its dispersal ranges have not been demonstrated in field studies.

Estimation of the precise dispersal range (mobility in a generation) of a given butterfly species is relatively complicated due to the spatial and temporal limitations of sampling, contingency of interactions between biotic and abiotic environments, and difficulty of distinguishing between individuals [37]. For the purposes of pest control, conservation, and understanding anthropogenic environmental stress, the marking-recapture method has been mainly employed for many years, but there are some inherent limitations to this method [38]; the values estimated based on this method are indirect and contain temporal uncertainty in the actual movement area of the organism. Therefore, a more direct estimation method is desirable whenever possible. In fact, harmonic radar tracking [39,40] or visual tracking [41,42] has successfully been employed to track butterflies in the field.

In this paper, we estimated the dispersal ranges of two subspecies of *Z. maha* in Japan using the direct estimation method of dispersal (i.e., the visual one-individual tracking method). This was possible simply because this butterfly does not have strong flying ability; it does not fly away quickly and does not fly high. Here, two populations were characterized in terms of “active” short dispersal. In contrast, recent northern range-margin expansion of this species [43,44,45] is likely driven by “passive” long dispersal by strong wind, which was beyond the scope of the present study. We quantitatively obtained dispersal-related and other fundamental behavioral data that may influence dispersibility indirectly. Comparisons were made between the two subspecies and between sexes. Based on these results, we discuss the feasibility of this species as an environmental indicator.

## 2. Materials and Methods

### 2.1. Measurement of Dispersal Range by the One-Individual Tracking Method

To obtain the original data for estimating the dispersal ranges of each subspecies of *Z. maha*, each individual was visually tracked in the field. Field work was conducted for *Z. m. okinawana* on November 22, 25, 26, and 29 and December 2, 2016, in Urasoe Sports Park, Urasoe City, Okinawa, Japan, or on the campus of the University of the Ryukyus, Nishihara Town, Okinawa, Japan, and for *Z. m. argia* on August 29, September 5, 26, and 29, and October 3 and 10, 2017, in Arakawa Sports Park, Toda City, Saitama, Japan. The environments of the sampling sites were all similar. They were open fields with lawns on the ground where colonies of *Oxalis* plants were found in patches. Some small flowers (mainly the family Asteraceae) and some trees (approximately 2–5 m in height) were also found. Before starting the tracking, environmental surveys were conducted at each sampling site on each day. The maximum temperature and the maximum wind speed within 30 s of the measurement period were measured using a digital temperature anemometer, the Smart Sensor AR816 (ARCO Science & Technology, Dongguan, China), and the maximum illumination of sunlight within 30 s was measured using a TM-205 Auto Ranging Light Meter (Tenmars Electronics, Taipei, Taiwan). One-individual tracking was conducted only on days when temperatures were in the range of 25.0–30.0 °C, the illuminance was over 35,000 lux, and the wind speed was under 2.0 m/s. These climate conditions meant that all field work was conducted only on calm, sunny (or sunny/cloudy) days when butterflies could freely fly.

A given individual was chosen at each sampling site, and the location data and behaviors of the butterfly were recorded until the individual was lost out of visual field or reduced its activities by sunset. Logging of the location data (latitude, longitude, and time) was conducted every 3 min using an M-241 wireless GPS location logger (Holux Technology, Hsinchu, Taiwan). When logging location data, the observer remained more than 1 m away from the butterfly in order not to disturb its behavior. Simultaneously, categories of behavior (duration of resting on leaves, rocks, or the ground, duration of feeding on flower nectar including species of these flowering plants, behavior of males chasing females, oviposition of females, and copulation-rejection behavior of females) were recorded. Although the location error of the GPS device was within 3 m according to the manufacturer’s specifications, each location data point was manually corrected, referring to the behavior records, when necessary before analysis, to estimate precise dispersal ranges.

Individuals who were tracked for 15 min or more were analyzed as valid samples. The numbers of valid samples were 13 for *Z. m. argia* males, 6 for *Z. m. argia* females, 8 for *Z. m. okinawana* males, and 4 for *Z. m. okinawana* females (*n* = 31 in total). The maximum tracking times were as follows: 126 min for *Z. m. argia* males, 183 min for *Z. m. argia* females, 150 min for *Z. m. okinawana* males, and 171 min for *Z. m. okinawana* females.

### 2.2. Comparison of Fundamental Behaviors of Each Subspecies

For a comparison of the behaviors of each subspecies, the following 12 dispersal-related and other behavioral traits were analyzed statistically within each sex: the mean distance of each recorded point from the first recorded (starting) point [m] (the distance from the starting point was obtained every 3 min until the tracked individual disappeared out of visual field or reduced its flight by sunset, the mean value of which was the mean distance for a given tracked individual); the mean flight speed of butterflies [m/min] (the flight speed for a given individual was calculated as follows: the distance from the previous point to the present point [m] divided by 3 [min]); the maximum flight speed [m/min] (the highest flight speed in the mean flight speed); accumulated distance in 10 days [km] (calculated from the mean flight speed); the frequency of resting [*n*/h]; the mean duration of resting [min]; the maximin duration of resting [min]; the frequency of feeding on flowers [*n*/h]; the proportion of time spent on the host plant (*Oxalis corniculata*) in feeding [%]; the frequency of mating behavior [*n*/h] (only in males); the frequency of rejection behavior of females to males [*n*/h] (only in females); and the frequency of oviposition [*n*/h] (only in females). For each trait, mean and standard deviation (SD) values were obtained irrespective of the normality or nonnormality of samples. We considered values of the mean ± 3SD (99.7% coverage) as the largest dispersal distance. All 12 categories were subjected to the Mann–Whitney *U* test using the free software R ver. 3.5.1 [46].

### 2.3. Estimation of Dispersal Distances by Statistical Prediction

For the estimation of the dispersal ranges of *Z. maha* in each subspecies, the ARIMA (autoregressive integrated moving average) model prediction was conducted using R [46] as above. The CRAN (Comprehensive R Archive Network) package used for this prediction was “forecast”. For the activity period of the adult butterflies, we assumed that butterflies were active for 12 h of daytime and inactive for 12 h of nighttime in a day, and the period of prediction time was set at 7200 min (10 days) after the tracking started based on the possible maximum lifespan of the adult butterfly [20]. For the best prediction, the best ARIMA model that was not a drifted model was selected based on the AIC (Akaike information criterion) values. We estimated 99% confidence interval (CI) values and considered them the largest dispersal distance. To assess the reliability of the models that were obtained, ME (mean error), RMSE (root mean squared error), and MAE (mean absolute error) were also calculated based on the models and compared to the values based on the raw (obtained) dataset.

## 3. Results

### 3.1. Dispersal-Related Traits

**Figure 1:**
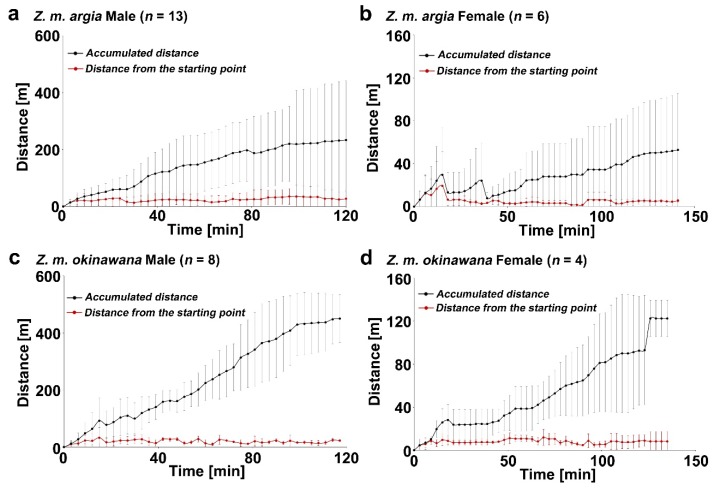
Dispersal distances of *Z. maha* males and females. Shown are the mean ± SD (standard deviation). The accumulated distance and the distance from the starting point were obtained using the number of individuals shown in each panel. A dot in late times does not necessarily represent the number of individuals shown in a panel because the tracking time varied among individuals. However, a dot represents at least two individuals. (**a**) *Z. m. argia* males. (**b**) *Z. m. argia* females. (**c**) *Z. m. okinawana* males. (**d**) *Z. m. okinawana* females.

**Figure 2:**
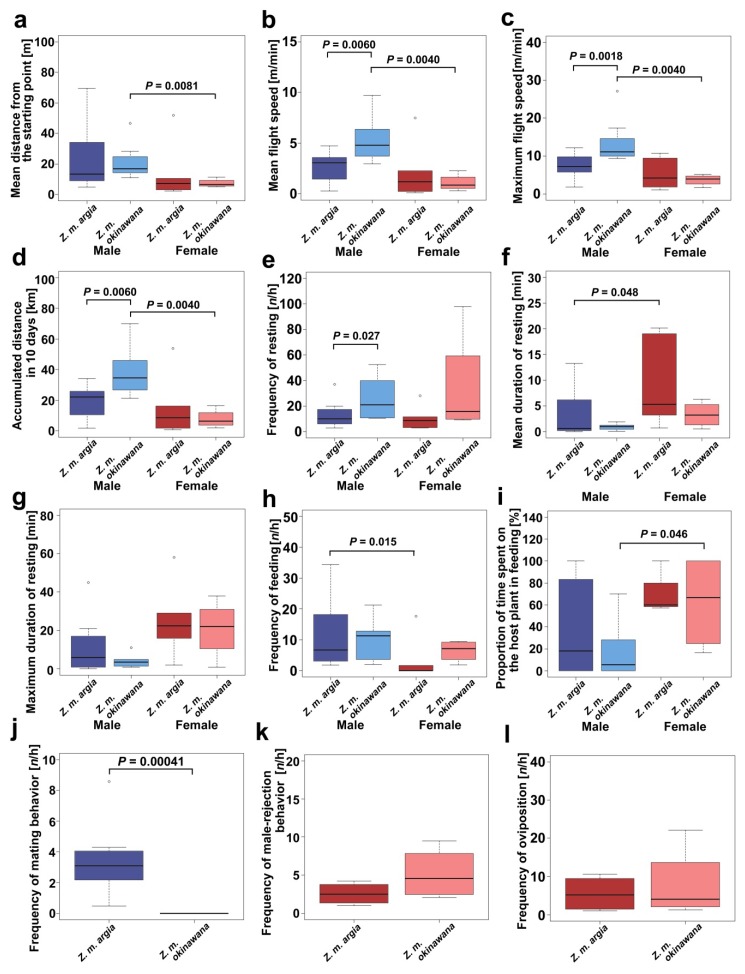
Comparison of 12 behavioral traits between subspecies and between sexes in *Z. maha*. (**a**) The mean distance from the starting point. (**b**) The mean flight speed. (**c**) The maximum flight speed. (**d**) The accumulated distance in 10 days. (**e**) The frequency of resting. (**f**) The mean resting duration. (**g**) The maximum resting duration. (**h**) The frequency of feeding. (**i**) The proportion of time spent on the host plant in feeding. (**j**) The frequency of mating behavior in males. (**k**) The frequency of male-rejection behavior in females. (**l**) The frequency of oviposition.

We first obtained two quantitative behavioral traits for the time dependence of distance directly from the observational data: the distance from the starting point and the accumulated distance (Figure 1). Although the accumulated distance of each subspecies increased with time, the mean distance of the recorded point from the starting point did not increase and was relatively stable, varying from the largest value, 24.2 ± 20.6 m for *Z. m. argia* males, to the smallest value, 7.4 ± 2.8 m for *Z. m. okinawana* females (Table 1). Accordingly, the mean + 3SD values for the mean distance from the starting point were calculated as follows: 86.0 m for *Z. m. argia* males, 55.9 m for *Z. m. okinawana* males, 70.4 m for *Z. m. argia* females, and 15.8 m for *Z. m. okinawana* females. The relatively small value in *Z. m. okinawana* females was notable. Comparisons of the mean distance from the starting point between subspecies showed no significant differences; *p* = 0.60 (in males) and *p* = 1 (in females) (Figure 2a; Table A1).

We also obtained the mean flight speed and the maximum flight speed, and we calculated the accumulated distance in 10 days (the adult lifespan) based on the mean flight speed (Table 1; Figure 2b–d). The mean flight speed of *Z. m. okinawana* males was statistically faster than that of *Z. m argia* males (*p* = 0.0060), but no significant difference was found between subspecies in females (*p* = 0.76) (Figure 2b; Table A1). The maximum flight speed was significantly faster in *Z. m. okinawana* than in *Z. m. argia* in males (*p* = 0.0018) (Figure 2c; Table A1). This result was reflected in the accumulated distance in 10 days; in males, *Z. m. okinawana* had a larger value than *Z. m. argia* (*p* = 0.0060) (Figure 2d).

### 3.2. Resting-Related and Feeding-Related Traits

We additionally obtained resting-related and feeding-related behavioral data in the two subspecies (Table 2). Most likely, because of the faster and longer flight shown above, *Z. m. okinawana* had a higher frequency of resting than *Z. m. argia* in males (*p* = 0.027) (Figure 2e; Table A1), although the mean resting duration (Figure 2f) and the maximum resting duration (Figure 2g) did not show significant differences between subspecies (Table A1). The frequency of feeding (Figure 2h) and the proportion of the host plant in feeding flowers (Figure 2i) did not show significant differences between subspecies (Table A1).

### 3.3. Sex-Related Traits

We further examined sex-related behavioral traits (Table 3). The frequency of mating behavior in males was significantly different between the two subspecies (Figure 2j; Table A1). Two female behavioral traits, the frequency of mate-rejection behavior and the frequency of oviposition, were not significantly different between the two subspecies (Figure 2k,l; Table A1).

### 3.4. Comparison between Sexes within Each Subspecies

In *Z. m. argia*, among the 12 categories, the mean duration of resting was higher in females (*p* = 0.048) (Figure 2f; Table A2). The frequency of feeding was higher in males (*p* = 0.015) (Figure 2h; Table A2). No other trait showed a statistically significant difference. Together, these results suggest that males are more active than females in *Z. m. argia*.

In *Z. m. okinawana*, the mean distance of recorded points from the starting point (*p* = 0.0081) (Figure 2a), the mean flight speed (*p* = 0.0040) (Figure 2b), the maximum flight speed (*p* = 0.0040) (Figure 2c), and the accumulated distance in 10 days (*p* = 0.0040) (Figure 2d) were significantly higher in males (Table A3). The proportion of the host plant in feeding flowers was higher in females than in males (*p* = 0.046) (Figure 2i). No significant difference was found in other traits. Together, these results suggest that males are more active than females in *Z. m. okinawana*, as in *Z. m. argia*. However, sexually “dimorphic” (significantly different) traits were not the same between the two subspecies.

### 3.5. Prediction of Dispersibility by the ARIMA (Autoregressive Integrated Moving Average) Model

Here, we obtained the predicted distance after 7200 min (10 days) from the starting point by the ARIMA model (Table 4). In *Z. m. argia*, the predicted distance of males was 52.3 m with 99% CI values of 0 to 706.6 m. In females, the predicted distance was 7.3 m with 99% CI values of 0 to 24.7 m. In *Z. m. okinawana*, the predicted distance of males was 18.6 m with 99% CI values of 3.0 to 34.1 m. In females, the predicted distance was 4.5 m with 99% CI values of 0 to 194.9 m. The high CI values likely resulted from individual variability in *Z. m. argia* males and *Z. m. okinawana* females.

In both subspecies and in both sexes, the absolute value of accuracy of these predictions by the ARIMA model were lower in all three categories (i.e., ME, RMSE, and MAE) than those of the raw input data (Table A4), justifying that the ARIMA models that were obtained here were reasonable.

## 4. Discussion

### 4.1. One-Individual Tracking Method in the Field: Advantages and Limitations

This paper is the first quantitative report on the dispersibility and associated behavioral traits of the pale grass blue butterfly in the field. Our one-individual tracking method is laborious but simple; one adult butterfly individual was visually tracked by an observer until it disappeared or stopped moving because of sunset. Importantly, the tracking data directly reflect the behaviors of butterflies in the field without any mathematical assumptions. This tracking study was possible because this species of butterfly flies slowly near the ground surface, and it is probably difficult, if not impossible, in most other species of butterflies in Japan. This slow and low flying near the ground surface is one of the reasons that this species has been used as an environmental indicator for radioactive pollution after the Fukushima nuclear accident in Japan.

In this paper, we focused on differences in two subspecies and sexes of this butterfly and made comparisons in dispersibility and its associated traits that were observed in the field. We observed local populations of *Z. m. argia* in Toda (Saitama Prefecture) and *Z. m. okinawana* in Urasoe and Nishihara (Okinawa Prefecture). We assumed in this study that quantitative differences in butterfly behaviors between these two observational localities represent differences between subspecies. However, we cannot exclude the possibility that our data may simply reflect behavioral traits of local populations and may not be generalizable to a species or subspecies. Indeed, the sampling sizes of this study were small, and the time of day for tracking varied.

All field work was conducted on calm days to estimate the ordinary state of mobility of this species. This means that the present study does not cover the possible dispersal ranges on windy days; if tracking was conducted on relatively windy days or areas, the dispersibility could become larger. However, we often observed in the field that flying individuals tended to swoop down on the ground when a sudden wind blew on a windy day, suggesting that the winds, if not very strong, might not considerably affect the dispersibility of individuals of this species. On the other hand, typhoon hits occur annually both in Okinawa and mainland Japan, which could mix populations.

### 4.2. Dispersal Ranges of the Pale Grass Blue Butterfly

With these limitations discussed above, we showed that the dispersal ranges of this butterfly were small. Simply because butterflies flew continuously, the accumulated distances increased over time, but importantly, the mean distances from the starting points were largely stable in both subspecies, indicating that butterflies fly around in a restricted small range.

The mean distances from the starting point, which were observational values, were largely consistent with the predicted distances, which were mathematically calculated values of the ARIMA model. For example, in *Z. m. okinawana* males, we obtained 21.1 m from observations (the mean distance) and 18.6 m from calculations (the ARIMA model). The observational and mathematical values were also similar in other subspecies and sexes. The largest dispersal distances based on the mean + 3SD were larger than those values above; for example, 86.0 m in *Z. m. argia* males. The largest dispersal distances based on the ARIMA 99% CI calculations were much larger; for example, 706.6 m in *Z. m. argia* males. On the basis of these results, it can be concluded that most butterflies found at a given site originated from that site itself. This is an important feature as an indicator species.

In previous studies, we focused on the biological effects of the Fukushima nuclear accident using the populations of *Z. maha* from seven localities around the Kanto-Tohoku districts (i.e., the cities and towns of Fukushima, Motomiya, Hirono, Iwaki, Takahagi, Mito, and Tsukuba). One important question was whether the individuals (populations) of these seven localities were reasonably isolated from one another or not. Considering the results of the present study, we can conclude that because the seven localities in the previous studies are more than 20 km apart from one another, butterfly individuals in these localities are highly difficult to intermix, at least within a single generation. However, gene flows among these localities are still possible after very strong winds or after many generations.

### 4.3. Differences between Subspecies

There was no statistically significant difference in the mean distance from the starting point between subspecies. On the other hand, the mean flight speed, the maximum flight speed, and the accumulated distance were significantly different between subspecies in males. It appears that *Z. m. okinawana* flies faster and longer in the accumulated distance. Perhaps because of active flight, *Z. m. okinawana* rests more frequently based on statistically significant differences between subspecies.

Despite the active flight in *Z. m. okinawana*, *Z. m. okinawana* males did not show any approaches to females in the field, although this may be due to the scarcity of females in the observational sites in Okinawa. This is in contrast to the relatively high frequency of mating behavior in *Z. m. argia* males. It appears that *Z. m. okinawana* males were less efficient than *Z. m. argia* in searching for females. Alternatively, there may be unknown functional aspects in their behaviors. At present, the higher flight activity in *Z. m. okinawana* than in *Z. m. argia* in males is not well understood.

The mean distance from the starting point was more variable (larger SD) in *Z. m. argia* than in *Z. m. okinawana* in males. Similarly, the predicted distance by the ARIMA model had larger 99% CI values in *Z. m. argia* than in *Z. m. okinawana* in males. The high variability in *Z. m. argia* means that some individuals in *Z. m. argia* likely had relatively high dispersibility, whereas other individuals did not fly so frequently. In contrast, in *Z. m. okinawana* males, all observed individuals flew relatively actively with relatively small SD and 99% CI values.

### 4.4. Differences between Sexes

Comparison of behavioral traits between sexes may indicate differences in strategies between sexes. In both subspecies, males were shown to be more active in flight than in females, which was suggested by larger values in males in the mean distance from the starting point, the mean flight speed, the maximum flight speed, and accumulated distance in 10 days, although these values were not significantly different in *Z. m. argia*. This sexual difference in flight has been known by researchers. Indeed, it is easier to find and catch males than females in the field. This study quantitatively confirmed this sexual difference in flight.

In *Z. m. argia*, significant sexual differences were detected in the mean duration of resting and the frequency of feeding. It may appear that males require occasional feeding more often than females because of higher activity in flight. However, in *Z. m. argia*, there was no significant difference in the mean distance from the starting point, mean flight speed, maximum flight speed, and accumulated distance in 10 days between sexes. In *Z. m. okinawana*, significant sexual differences were detected in the mean distance from the starting point, the mean flight speed, the maximum flight speed, the accumulated distance in 10 days, and the proportion of the host plant in feeding. However, the frequency of resting was not significant. Thus, significance in the frequency of resting in *Z. m. argia* between sexes cannot be explained by higher activity in flight in males. It should be noted that different sets of quantitative traits were significant in the two subspecies. This finding may suggest that sexual strategies are different between subspecies.

### 4.5. Comparisons with Other Butterflies

According to the ARIMA results, the dispersal ranges of this species may be considered up to 706.6 m in *Z. m. argia* males. This is the largest value among subspecies and sexes examined in this study. The larger dispersal ranges in males are consistent with studies of other butterflies [47] and fruit flies [5], in which males have higher dispersibility than females. These results indicate that individuals belonging to two different populations that are more than several hundred meters apart from each other are difficult to intermix, at least within a single generation. This spatial isolation of populations at least within a generation may not be enough to isolate populations and drive evolution, but this partial spatial isolation would be sufficient for this species to be qualified as an environmental indicator.

The dispersal ranges that were obtained in this study may be compared with those of other butterfly species. In the cabbage white butterfly *Pieris rapae*, it was reported using the mark-recapture method that the mean distance of the recapture point from the release point was estimated to be approximately 330 m [48]. In the Japanese large blue butterfly *Shijimiaeoides divinus*, the maximum distance of the recapture point from the release point was estimated to be 560 m [49]. Note that these species are larger in body size than the pale grass blue butterfly. The Glanville fritillary butterfly *Melitaea cinxia* had a maximum recapture distance of 1150 m [50]. Note that this is a nymphalid butterfly that has a much stronger flying ability than the pale grass blue butterfly. In contrast, in the case of the large blue butterfly, *Phengaris arion* (*Maculinea arion*), over 85% of recaptured individuals were within 5.7 km and that gene flow can occur at a distance of 90 km apart by the contributions of very rare long-distance dispersal individuals [51]. Therefore, the pale grass blue butterfly has a reasonable dispersal range among these butterflies. Additionally, it is reported in *Pieris rapae* that females change their behavior by time of day and that the dispersibility of each individual can change by age in days after eclosion [52]. Molecular analyses of the genetic structure of the population and field studies on time-dependent and age-dependent changes in dispersal ranges may help to more precisely understand the ecological population structures of *Z. maha*.

### 4.6. Dispersal of Northern Range-Margin Populations

The pale grass blue butterfly has been known to expand its range margins to the north since the beginning of this century in the northwestern Tohoku district, Japan [43,44,45]. This range expansion over many generations was associated with remarkable color-pattern modifications of this species [43,44,45], and these observations together with laboratory studies have been considered an important demonstration of genetic assimilation in the field [53]. In that case, relatively long-range dispersal by a small number of dispersers over many generations, which is beyond the short-range dispersal in a single generation shown in the present study, might have occurred to expand the range margins. This range-margin expansion is likely driven by “passive” dispersal by strong wind because the “active” dispersal distances of this species are small, as shown in this study.

Dispersal mechanisms over generations in the field may be more complex than a simple accumulation of short-term short-range dispersal in a single generation that was observed in the present study. Further investigations on passive long-term long-range dispersal in multiple generations are expected in the future.

## 5. Conclusions

In this study, we determined the dispersal ranges of the pale grass blue butterfly in the field by the one-individual tracking method. The dispersal ranges of this butterfly were small, despite some variation among subspecies and sexes. Some behavioral traits of this butterfly in the field were different between the two subspecies of this butterfly, but their functional significance is unclear. These results justify that both subspecies of this butterfly in Japan are suitable for an environmental indicator for a given locality because of the scarcity of migrating individuals.

## Figures and Tables

**Table 1:** Dispersal-related traits in the two subspecies of *Z. maha*.

Subspecies	Sex	Number of Individuals Tracked [*n*]	Mean Distance of Recorded Points from the Starting Point [m]	Mean Flight Speed [m/min]	Maximum Flight Speed [m/min]	Accumulated Distance in 10 Days (Calculated from the Mean Flight Speed) [km]
*Z. m. argia*	Male	13	24.2 ± 20.6	2.6 ± 1.4	7.3 ± 3.1	18.9 ± 10.2
	Female	6	13.7± 18.9	2.1 ± 2.8	5.3 ± 4.1	15.0 ± 20.3
*Z. m. okinawana*	Male	8	21.1 ± 11.6	5.3 ± 2.3	13.5 ± 6.1	38.2 ± 16.3
	Female	4	7.4 ± 2.8	1.1 ± 0.8	3.7 ± 1.5	7.8 ± 6.1

Note: Numbers show the mean ± standard deviation.

**Table 2:** Resting-related and feeding-related behaviors in the two subspecies of *Z. maha*.

Subspecies	Sex	Frequency of Resting [*n*/h]	Mean Duration of Resting [min]	Maximum Duration of Resting [min]	Frequency of Feeding [*n*/h]	Proportion of the Host Plant in Feeding Flowers [%]
*Z. m.* *argia*	Male	12.9 ± 9.3	3.3 ± 4.5	10.0 ± 12.8	12.0 ± 12.8	43.7 ± 45.5
	Female	10.6 ± 9.3	9.0 ± 8.5	25.0 ± 19.0	9.6 ± 11.3	72.4 ± 24.0
*Z. m.* *okinawana*	Male	25.8 ± 16.3	0.9 ± 0.6	4.0 ± 3.3	9.8 ± 6.4	17.2 ± 25.4
	Female	34.6 ± 42.5	3.3 ± 2.5	20.8 ± 15.3	6.4 ± 3.5	62.5 ± 43.8

Note: Numbers show the mean ± standard deviation.

**Table 3:** Frequency of sex-related behaviors in the two subspecies of *Z. maha*.

Subspecies	Sex	Frequency of Mating Behavior in Males [*n*/h]	Frequency of Mate-Rejection Behavior in Females [*n*/h]	Frequency of Oviposition [*n*/h]	Estimated Number of Eggs Laid by a Female in 10 Days [*n*]
*Z. m.* *argia*	Male	3.5 ± 2.4	-	-	-
	Female	-	5.5 ± 4.7	2.3 ± 3.5	657 ± 565
*Z. m.* *okinawana*	Male	0.0	-	-	-
	Female	-	5.2 ± 3.4	7.9 ± 9.6	944 ± 1155

Note: Numbers show the mean ± standard deviation.

**Table 4:** Predicted distance from the starting point after 7200 min by the ARIMA (autoregressive integrated moving average) model.

Subspecies	Sex	Predicted Distance from the Starting Point [m]	99% Confidence Interval [m]		AIC
			Upper Bound	Lower Bound	
*Z. m. argia*	Male	52.3	706.6	< 0 (−602.1)	259.1
	Female	7.3	24.7	< 0 (−10.2)	334.1
*Z. m. okinawana*	Male	18.6	34.1	3.0	329.1
	Female	4.5	194.9	< 0 (−185.9)	229.6

## Appendix A

Appendix tables (Table A1, Table A2, Table A3 and Table A4) are shown below. Table A1, Table A2 and Table A3 show the results of the *U*-tests described in the Results section. Table A4 is associated with the ARIMA model.

**Table A1:** Values of the *U* test between the two subspecies of *Z. maha*.

	Sex	*W*	*p*-Value
Mean distance of recorded points from the starting point	Male	44	0.60
	Female	12	1
Mean flight speed	Male	15	0.0060
	Female	10	0.76
Maximum flight speed	Male	11	0.0018
	Female	14	0.76
Accumulated distance in 10 days	Male	15	0.0060
	Female	10	0.76
Frequency of resting	Male	21	0.027
	Female	6	0.26
Mean duration of resting	Male	53.5	0.94
	Female	17	0.35
Maximum duration of resting	Male	62.5	0.47
	Female	13	0.91
Frequency of feeding	Male	33	0.81
	Female	4	0.099
Proportion of the host plant in feeding	Male	49	0.21
	Female	7	0.85
Frequency of mating behavior in males	Male	64	0.00041
	Female	-	-
Frequency of mate-rejection behavior in females	Male	-	-
	Female	4	0.34
Frequency of oviposition	Male	-	-
	Female	7	0.89

**Table A2:** Values of the *U* test between sexes in *Z. m. argia*.

	*W*	*p*-Value
Mean distance of recorded points from the starting point	58	0.11
Mean flight speed	57	0.13
Maximum flight speed	51	0.32
Accumulated distance in 10 days	57	0.13
Frequency of resting	47	0.51
Mean duration of resting	16	0.048
Maximum duration of resting	18.5	0.078
Frequency of feeding	48	0.015
Proportion of the host plant in feeding	9	0.45

**Table A3:** Values of the *U* test between sexes in *Z. m. okinawana.*

	*W*	*p*-Value
Mean distance of recorded points from the starting point	31	0.0081
Mean flight speed	32	0.0040
Maximum flight speed	32	0.0040
Accumulated distance in 10 days	32	0.0040
Frequency of resting	18	0.81
Mean duration of resting	6	0.11
Maximum duration of resting	7	0.15
Frequency of feeding	23	0.28
Proportion of the host plant in feeding	4	0.046

**Table A4:** Accuracy of the ARIMA prediction of each subspecies.

Subspecies	Sex	Dataset	ME	RMSE	MAE
*Z. m. argia*	Male	Prediction	1.22	5.10	3.13
		Raw	-26.9	28.3	26.9
	Female	Prediction	0.12	3.32	1.91
		Raw	-2.13	6.75	4.52
*Z. m. okinawana*	Male	Prediction	0.089	5.74	4.52
		Raw	0.10	6.02	4.80
	Female	Prediction	0.080	1.60	1.16
		Raw	2.43	3.57	3.00
